# Joint effect of water and sanitation practices on childhood diarrhoea in sub-Saharan Africa

**DOI:** 10.1371/journal.pone.0283826

**Published:** 2023-05-11

**Authors:** Iddrisu Amadu, Abdul-Aziz Seidu, Kwabena Koforobour Agyemang, Francis Arthur-Holmes, Eric Duku, Iddrisu Salifu, Obasanjo Afolabi Bolarinwa, John Elvis Hagan Jr., Bright Opoku Ahinkorah

**Affiliations:** 1 Africa Centre of Excellence in Coastal Resilience, University of Cape Coast, Cape Coast, Ghana; 2 Department of Fisheries and Aquatic Sciences, School of Biological Sciences, College of Agriculture and Natural Sciences, University of Cape Coast, Cape Coast, Ghana; 3 Emperiks Research, Tamale, Ghana; 4 Department of Population and Health, College of Humanities and Legal Studies, University of Cape Coast, PMB, Cape Coast, Ghana; 5 College of Public Health, Medical and Veterinary Sciences, James Cook University, Townsville, Queensland, Australia; 6 Department of Estate Management, Takoradi Technical University, Takoradi, Ghana; 7 Department of Geography and Regional Planning, University of Cape Coast, Cape Coast, Ghana; 8 Department of Sociology and Social Policy, Lingnan University, Tuen Mun, Hong Kong; 9 Hen Mpoano (Our Coast), Sekondi-Takoradi, Ghana; 10 Department of Public Health Medicine, School of Nursing and Public Health, University of KwaZulu-Natal, Durban, South Africa; 11 Department of Public Health & Well-being, Faculty of Health & Social Care, University of Chester, Chester, United Kingdom; 12 Department of Health, Physical Education, and Recreation, University of Cape Coast, Cape Coast, Ghana; 13 Neurocognition and Action-Biomechanics-Research Group, Faculty of Psychology and Sport Sciences, Bielefeld University, Bielefeld, Germany; 14 School of Public Health, Faculty of Health, University of Technology, Sydney, NSW, Australia; Gadjah Mada University Faculty of Medicine, Public Health, and Nursing: Universitas Gadjah Mada Fakultas Kedokteran Kesehatan Masyarakat dan Keperawatan, INDONESIA

## Abstract

**Background:**

Diarrhoea remains the major cause of morbidity and mortality of children under five years in Africa. Several studies have shown that inadequate and unsafe water, lack of sanitation, and poor hygiene practices are complex issues for different pathogens and accountable for the occurrence of diarrhoea diseases. We assessed the combined effect of household’s source of drinking water and type of toilet facility and residential wellbeing on the incidence of childhood diarrhoea in 33 Sub-Saharan Africa countries while accounting for relevant compositional and contextual factors.

**Methods:**

The 2010–2019 datasets from the Demographic and Health Surveys were drawn for analyses. The outcome variable used in this study was the incidence of childhood diarrhoea. Three negative log-log generalized linear regression models were then sequentially fitted to the data to examine the joint effect of household water and sanitation practices on child diarrhoea. The results were presented using crude odds ratios (CORs) and adjusted odds ratios (AORs) at 95% confidence intervals (CIs). Using ArcGIS software, maps were design to unveil the spatial distribution of key variables.

**Findings:**

Approximately 16% of the 307,741 mothers interviewed reported an incidence of diarrhoea disease among children under-five years in their households. The results showed that a household depending on an unimproved source of drinking water and with an unimproved type of toilet facility was not significantly associated with childhood diarrhoea. However, those with improved drinking water but an unimproved type of toilet facility had higher odds of reporting childhood diarrhoea (AOR = 1.020, 95% CI = 1.003-1-036) compared to those in households with both improved source of drinking water and type of toilet facility. Across the geographical regions, Eastern (aOR = 1.102, 95% CI = 1.084–1.120) and Central Africa (aOR = 1.102, 95% CI = 1.083–1.121) were more likely to experience child diarrhoea.

**Conclusion:**

Water and sanitation practices such as the source of drinking water and toilet facility, and geographic region had significant effects on childhood diarrhoea in sub-Saharan Africax. The findings suggest the need for multi-sectoral actions that recognise the geo-spatial and temporal characteristics identified in the study through regional to national policies. Water and sanitation community-based interventions that seek to improve equitable access to safe water and sanitation in the sub-region should be intensified.

## Background

Access to safe drinking water, sanitation facilities and proper hygiene is fundamental to the maintenance of good health and wellbeing. Improving access to safe water, adequate sanitation and proper hygiene are on the frontline of efforts to achieve the anticipated 2030 Sustainable Development Goals 3 and 6 [1]. However, unclean drinking water and poor sanitation conditions remain a big issue of concern in public health [1, 2]. Despite progress in water, and sanitation, many children under five years old continue to die around the world [1]. According to the World Health Organisation (WHO), unclean drinking water and poor sanitation are the major causes of child mortality, with diarrhoea as one of the leading causes [1]. Unsafe water and poor sanitation still account for 842,000 diarrhoea deaths every year, and constrain effective prevention and management of other diseases including malnutrition, non-transmissible diseases (NTDs), and cholera [1].

A recent report from the United Nations Inter-agency Group for Child Mortality Estimation (UN-IGME) indicates that 6.3 million children and young adolescents died in 2017 alone [2]. Of these, 5.4 million were children under five, with 2.5 million deaths occurring in the first month of life, 1.6 million in 1–11 months, and 1.3 million between 1 and 4 years [2]. In Africa, the incidence of diarrhoea diseases associated with unclean water and poor sanitation remains high and they are considered one of the leading causes of childhood mortality, especially, in sub-Saharan countries [3].

Studies on the effects of Water Sanitation and Hygiene (WASH) interventions on children’s diarrhoea status show that inadequate and potable water, lack of sanitation, and poor hygiene practices create favourable conditions for pathogens (such as Escherichia coli, Shigella, Campylobacter, etc), increasing the risk of diarrhoea diseases, although they have also shown the importance of environmental factors in childhood diarrhoea prevalence [4]. Other studies have also reported a positive association between childhood diarrhoea prevalence and access to improved drinking water sources as well as toilet facilities [4–6].

The different conclusions drawn from the above studies may have resulted from the differences in underlying contextual factors, such as social and environmental conditions. Since most studies were conducted at the individual country level, the extent of the impacts from these facilities may vary, possibly because of context. However, while substantial evidence on the independent effects of water and sanitation at the various country-levels exists, the cumulative joint effect of water and sanitation on child diarrhoea remains unexplored. Afitiri et al. [7], for example, assessed the combined effects of environmental factors on childhood diarrhoea prevalence and morbidity over 21 years, focusing on Ghana. However, the literature on childhood diarrhoea does not explicitly capture the joint relationship between water and sanitation on childhood diarrhoea status in sub-Saharan Africa (SSA).

The aim of this study, therefore, was to contribute towards bridging the gap in the literature by investigating the combined effects of environmental factors on childhood diarrhoea prevalence and morbidity in SSA. A comprehensive study assessing the contributions of environmental, compositional, and contextual factors to diarrhoea prevalence among children in SSA is necessary to reveal the commonalities at the national and sub-national scales, to inform policy and intervention design.

Extending from Afitiri et al’s [7] work, this study was used to assess the combined effects of drinking water source, and type of toilet facility and residential wellbeing, on childhood diarrhoea and morbidity prevalence in 33 sub-Saharan African countries, while accounting for relevant compositional and contextual factors. The major research issues addressed include: the general trend of diarrhoea prevalence among children under five years of age in SSA between 2010 and 2019; the cumulative effects of drinking water source, and toilet facility and residential wellbeing on diarrhoea prevalence amongst children under five years; and the order of magnitude of compositional and contextual factors that influence diarrhoea prevalence amongst children under five years.

### Methods

#### Study design and data sources

**Fig 1:**
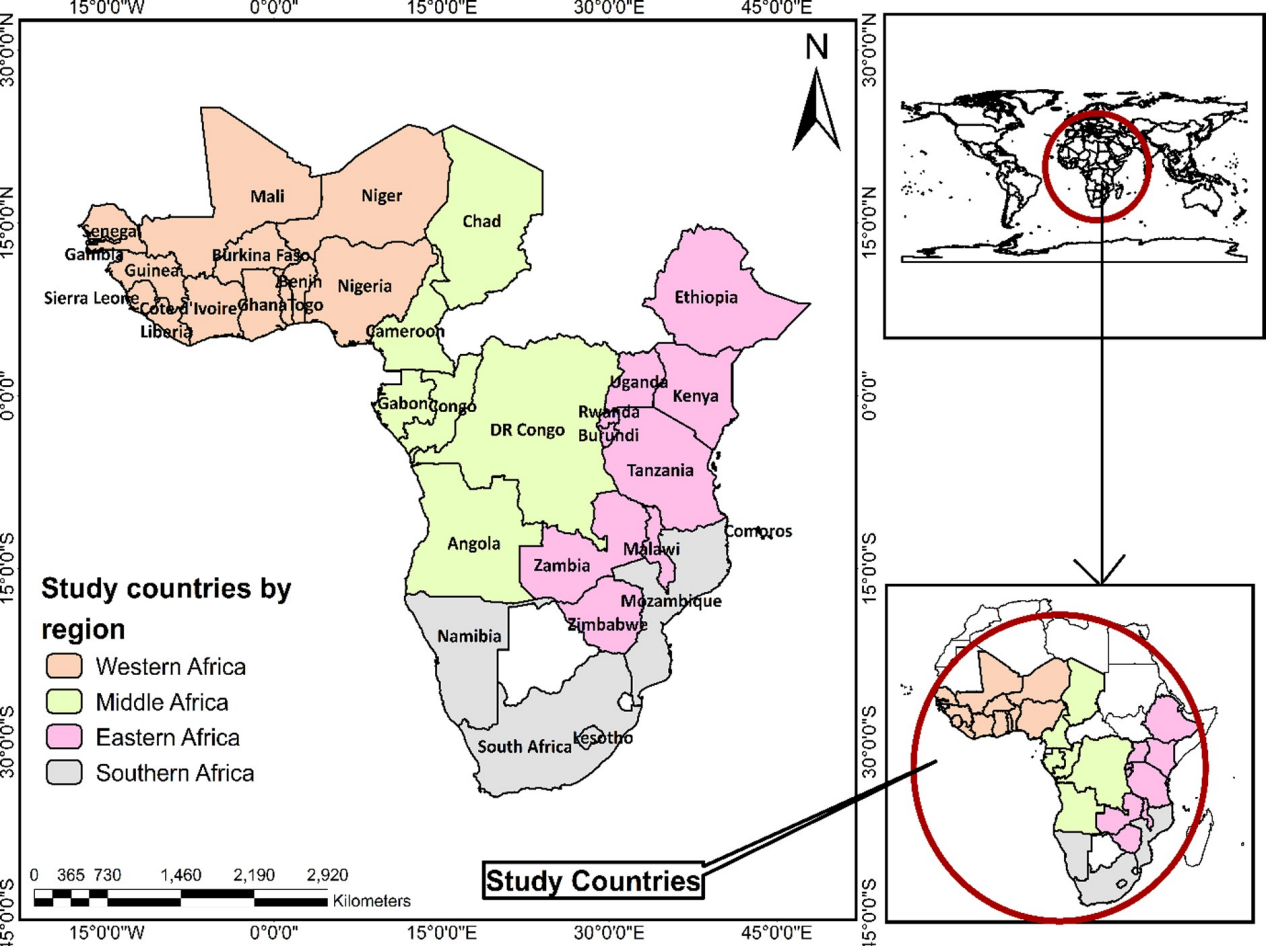
The 33 sub-Saharan African countries considered. Republished from EfrainMaps, under a CC BY license, with permission from EfrainMaps, original copyright 2022. Note: This map was constructed by Authors based on shapefiles freely downloaded from EfrainMaps website (https://tapiquen-sig.jimdofree.com/descargas-gratuitas/mundo/).

The 2010–2019 datasets from 33 sub-Saharan African countries from the Demographic and Health Surveys (DHS) were used. The DHS Program provides nationally representative datasets from surveys conducted in over 90 low-and middle-income countries around the world. Since 1984, more than 400 cross-sectional surveys have been conducted using standardised protocols to collect, analyse and disseminate data on critical population and health indicators, and health and socio-demographic household characteristics, etc. Standardised protocols, including instruments and ethics, are used across the countries surveyed to enable multi-country analyses and data comparison. Data are gathered from respondents using a two-stage, stratified, sampling procedure. Datasets are made available at https://dhsprogram.com/data/available-datasets.cfm upon approval of a formal request. The geographical information in the form of shapefiles indicating the boundaries of countries is freely downloaded from the EfrainMaps’ website (https://tapiquen-sig.jimdofree.com/descargas-gratuitas/mundo/). The countries included in this study are depicted in Fig 1. Permission to use the shapefiles has been attached as a S1 Fig.

### Variables studied

#### Outcome

The outcome variable is the incidence of diarrhoea in children under five years. This was measured by asking the respondent if the child had diarrhoea recently and dichotomized with response categories “Yes” (= 1) and “No” (0). Missing responses were dropped using listwise deletion.

### Main explanatory variable

The main explanatory variable was “water and sanitation practices” derived from the household’s source of drinking water and type of toilet facility. Following the WHO/UNICEF Joint Monitoring Programme (JMP) classification of household water and sanitation services, these variables were dichotomised, individually, with response categories “improved” and “unimproved”–see Table 1 [7, 8].

**Table 1:** WHO/UNICEF JMP 2017 categorisation of households’ source of drinking water and type of toilet facility [7].

Service	Category	
	Improved	Unimproved
Source of drinking water	Boreholes or tube-wells, protected springs, piped water, protected dug wells, packaged or delivered water, and rainwater.	Pond, stream, dam, canal, unprotected dug well, river, unprotected spring, canal and irrigation, and lake.
Type of toilet facility	Septic tanks or pit latrines; flush/pour flush, piped sewer systems, composting toilets or pit latrines with slabs, and ventilated improved pit latrines.	Open defecation, hanging latrines or bucket latrines, and pit latrines without a slab or platform.

The variable “water and sanitation practices” was derived following Afitiri et al. [7]. The four response categories were:

“Improved-improved” households with improved drinking water source and toilet facilities;
“Improved-unimproved” households with improved drinking water source and unimproved toilet facilities);
“Unimproved-improved” households with unimproved drinking water source and improved toilet facilities); and
“Unimproved-unimproved” households with unimproved drinking water source and unimproved toilet facility.

The aim was to understand the joint effect of unimproved water and sanitation practices on child diarrhoea in SSA. “Improved-improved” was used as the reference category.

### Control variables

The effects of relevant individual-level (child, mother, and household head) characteristics, referred to as “biosocial factors” and household-level (socio-cultural) factors, which together make up “compositional factors”, were adjusted for in the multivariable statistical procedure [7]. The effect of place-based factors, referred to as “contextual factors”, was also controlled for. The compositional variables included the current age of the child in years (up to and including 4); sex of the child (male/female); birth order; mother’s age in groups (15–19, 20–24, 25–29, 30–34, 35–39, 40–44 and 45–49); mother’s highest education level (no formal education, primary, secondary and higher); and partner/husband’s highest education level (no formal education, primary, secondary and higher). Other compositional factors were antenatal visits during pregnancy (no/yes); postnatal check within 2 months (no/yes); place of child’s delivery (home, health facility, and other); sex of household head (male/female); and age of household head (young-adult (below 35); middle-aged (35 to 55) and old-aged (over 55 [see [9]]. Household size (small, medium, large); access to electricity (no/yes); and wealth status (poor, middle, rich) were the remining compositional factors. The contextual factors considered were place of residence (urbanicity) with response categories “urban” and “rural”, and geographical regions.

### Statistical analyses

Stata SE version 14.2 software (StataCorp, texas, USA) was used for the statistical analyses in this study. To minimise the potential effects of multiple sampling techniques, the data were first declared “survey data” using the “svyset” command, with sample weight and stratification variables. Descriptive statistics (weighted percentages and frequencies) were used to present the distributions of the variables considered. Bivariate analyses using the chi-square test of independence were conducted to assess the associations between the compositional and contextual variables and the incidence of childhood diarrhoea. Explanatory variables were assessed for multicollinearity and there was no evidence of multicollinearity (mean VIF = 1.47; maximum VIF = 2.20; minimum VIF = 1.00). Three negative log-log generalised linear regression models were then fitted sequentially to the data to examine the joint effects of household water and sanitation practices on childhood diarrhoea. The first model (Model 1) constituted the joint effect of household water and sanitation practices on childhood diarrhoea. The second (Model 2) and third (Model 3) models adjusted, respectively, for the effects of the relevant compositional and contextual variables considered. Before fitting all models, the cluster variable was specified to control for the effect of clustering in the data. The results were presented using crude odds ratios (CORs) and adjusted odds ratios (AORs) at 95% confidence intervals (CIs). Robust standard errors (Robust SEs) were also ascertained by incorporating the cluster and sample weights in the models to adjust for potential effects of the complex survey design. The weighted percentage of the variables ‘‘incidence of childhood diarrhoea” and “water and sanitation practices” for each of the study countries in Excel format were imported to ArcMap 10.7. The imported weighted percentages of the two variables were joined to the shapefiles for the 33 countries using the spatial join technique. Based on the output future class from the spatial join, maps with graduated colours were created to spatially unveil the distribution of incidence of child diarrhoea across countries and regions using the group analysis spatial statistics tool in ArcMap 10.7. A map with pie chart symbols was also created to show the proportion of source of drinking water and type of toilet facility for each region, as a categorical variable, using the ArcMap 10.7. The spatial autocorrelation (Moran’s I) tool in ArcMap 10.7 was used to assess whether distribution of diarrhoea among under five children across the study countries is either random, dispersed, or clustered.

Following the generalised linear function:

$$g(\mu i)=\eta \underline{i}=\alpha +\beta 1X\;i1+\beta 2X\;i2+\cdots +\beta \;k\;X\;ik$$

Applying the linearised link function (-log-log) yields

−loge[−loge(μi)]=α+β1Xi1+β2Xi2+⋯+βkXik

Where μi = outcome variable (incidence of diarrhoea

α = intercept

β1…. β k = Coefficients

X i1…. X ik = Explanatory variables

Model 1 (Kay predictor) could be specified as:

$\begin{array}{l} -\text{loge}[-\text{loge}(\text{incidence}\;\text{of}\;\text{diarrhoea})\;]\\ =\alpha +\beta 1\;(\text{Source}\;\text{of}\;\text{drinking}\;\text{water-type}\;\text{of}\;\text{toilet}\;\text{facility}) \end{array}$

Model 2 (Key predictor +Compositional factors):

$$\begin{array}{l} \text{-loge}\left[\text{-loge}\left(\text{incidence}\;\text{of}\;\text{diarrhoea}\right)\right]=\alpha +\beta 1\left(\text{Source}\;\text{of}\;\text{drinking}\;\text{water-type}\;\text{of}\;\text{toilet}\;\text{facility}\right)\\ +\beta 2\left(\text{current}\;\text{age}\;\text{of}\;\text{child}\right)+\beta 3\left(\text{Sex}\;\text{of}\;\text{child}\right)\;\\ +\beta 4\left(\text{Birth}\;\text{order}\right)+\beta 5\left(\text{Age}\;\text{of}\;\text{mother}\right)+\beta 6\left(\text{Mother}’\text{s}\;\text{highest}\;\text{education}\right)\;\\ +\beta 7\left(\text{Antenatal}\;\text{visits}\;\text{during}\;\text{pregnancy}\right)+\beta 8\left(\text{postnatal}\;\text{check}\;\text{within}\;\text{2months}\right)\\ +\beta 9\left(\text{Place}\;\text{of}\;\text{delivery}\right)+\beta 10\left(\text{Partner/husband}’\text{s}\;\text{age}\right)+\beta 11\left(\text{Sex}\;\text{of}\;\text{household}\;\text{head}\right)\\ +\beta 12\left(\text{Age}\;\text{of}\;\text{household}\;\text{head}\right)+\beta 13\left(\text{Household}\;\text{size}\right)+\beta 14\left(\text{Access}\;\text{to}\;\text{electricity}\right)\\ +\beta 15\left(\text{Wealth}\;\text{status}\right). \end{array}$$

Model 3 (Key predictor + compositional factors+ contextual factors)

$$\begin{array}{l} \text{-loge}\left[\text{-loge}\left(\text{incidence}\;\text{of}\;\text{diarrhoea}\right)\right]=\alpha +\beta 1\left(\text{Source}\;\text{of}\;\text{drinking}\;\text{water-type}\;\text{of}\;\text{toilet}\;\text{facility}\right)\\ +\beta 2\left(\text{current}\;\text{age}\;\text{of}\;\text{child}\right)+\beta 3\left(\text{Sex}\;\text{of}\;\text{child}\right)+\beta 4\left(\text{Birth}\;\text{order}\right)+\beta 5\left(\text{Age}\;\text{of}\;\text{mother}\right)\\ +\beta 6\left(\text{Mother}’\text{s}\;\text{highest}\;\text{education}\right)+\beta 7\left(\text{Antenatal}\;\text{visits}\;\text{during}\;\text{pregnancy}\right)\\ +\beta 8\left(\text{postnatal}\;\text{check}\;\text{within}\;\text{2months}\right)+\beta 9\left(\text{Place}\;\text{of}\;\text{delivery}\right)+\beta 10\left(\text{Partner/husband}’\text{s}\;\text{age}\right)\\ +\beta 11\left(\text{Sex}\;\text{of}\;\text{household}\;\text{head}\right)+\beta 12\left(\text{Age}\;\text{of}\;\text{household}\;\text{head}\right)+\beta 13\left(\text{Household}\;\text{size}\right)\\ +\beta 14\left(\text{Access}\;\text{to}\;\text{electricity}\right)+\beta 15\left(\text{Wealth}\;\text{status}\right)+\beta 16\left(\text{Urbanicity}\right)+\beta 17\left(\text{Geographical}\;\text{region}\right)\text{.} \end{array}$$

### Ethics

The Inner-City Fund International Review Board evaluated and approved the techniques and questionnaires used in the DHS data. All survey participants provided informed consent and permission to use the data anonymously, which the enumerators obtained. DHS surveys follow ethical guidelines and are compliant with US government health and service requirements. The DHS datasets are freely available online. The data used for the study were obtained from the Demographic and Health Surveys (DHS) Program. The dataset is available at: http://goo.gl/ny8T6X.

## Results

### Prevalence of childhood diarrhoea and joint water and sanitation practices

**Fig 2:**
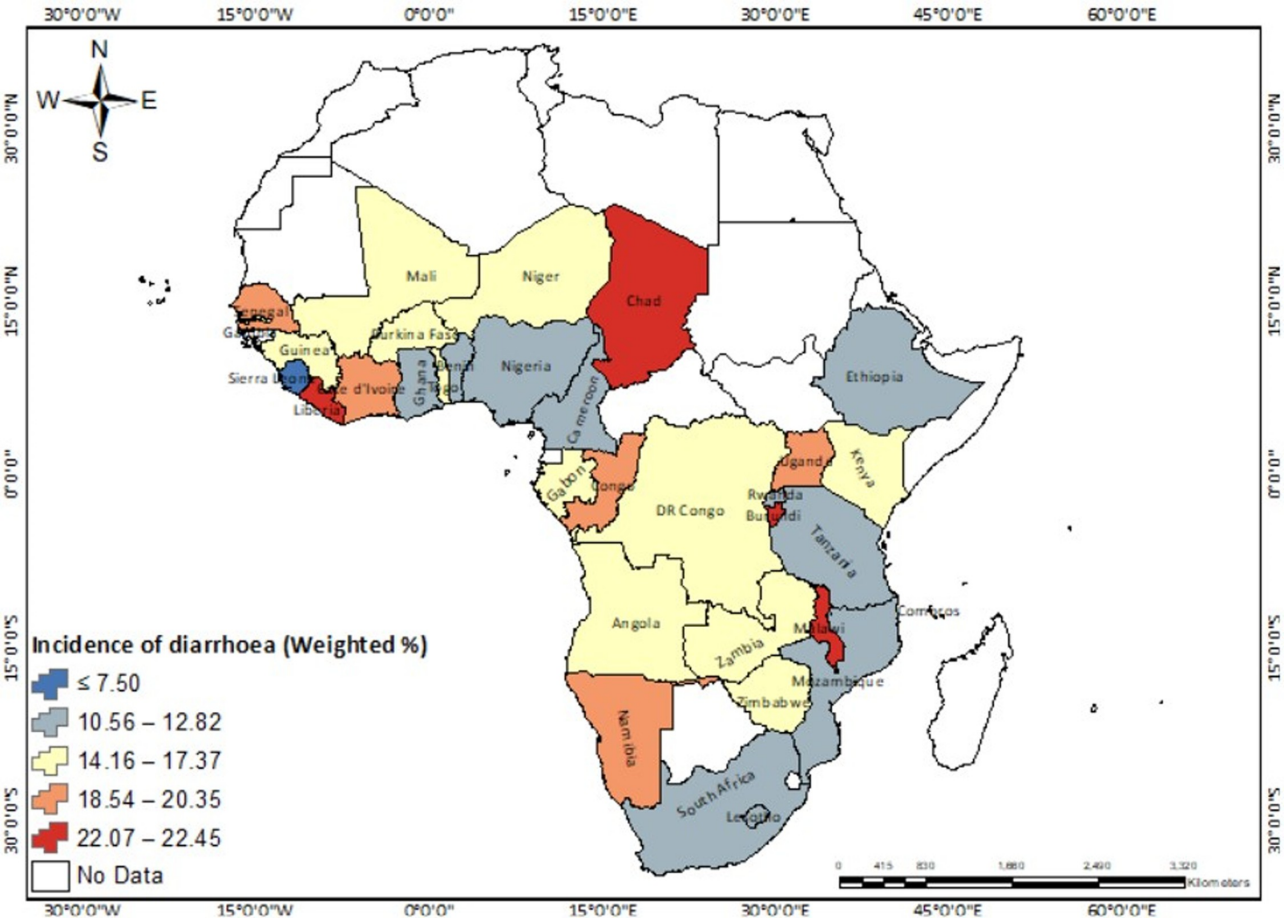
Incidence of diarrhoea among children under five in 33 SSA countries. Republished from EfrainMaps, under a CC BY license, with permission from EfrainMaps, original copyright 2022. Note: Authors’ construct based on shapefiles freely downloaded from EfrainMaps website (https://tapiquen-sig.jimdofree.com/descargas-gratuitas/mundo/) and DHS data.

**Fig 3:**
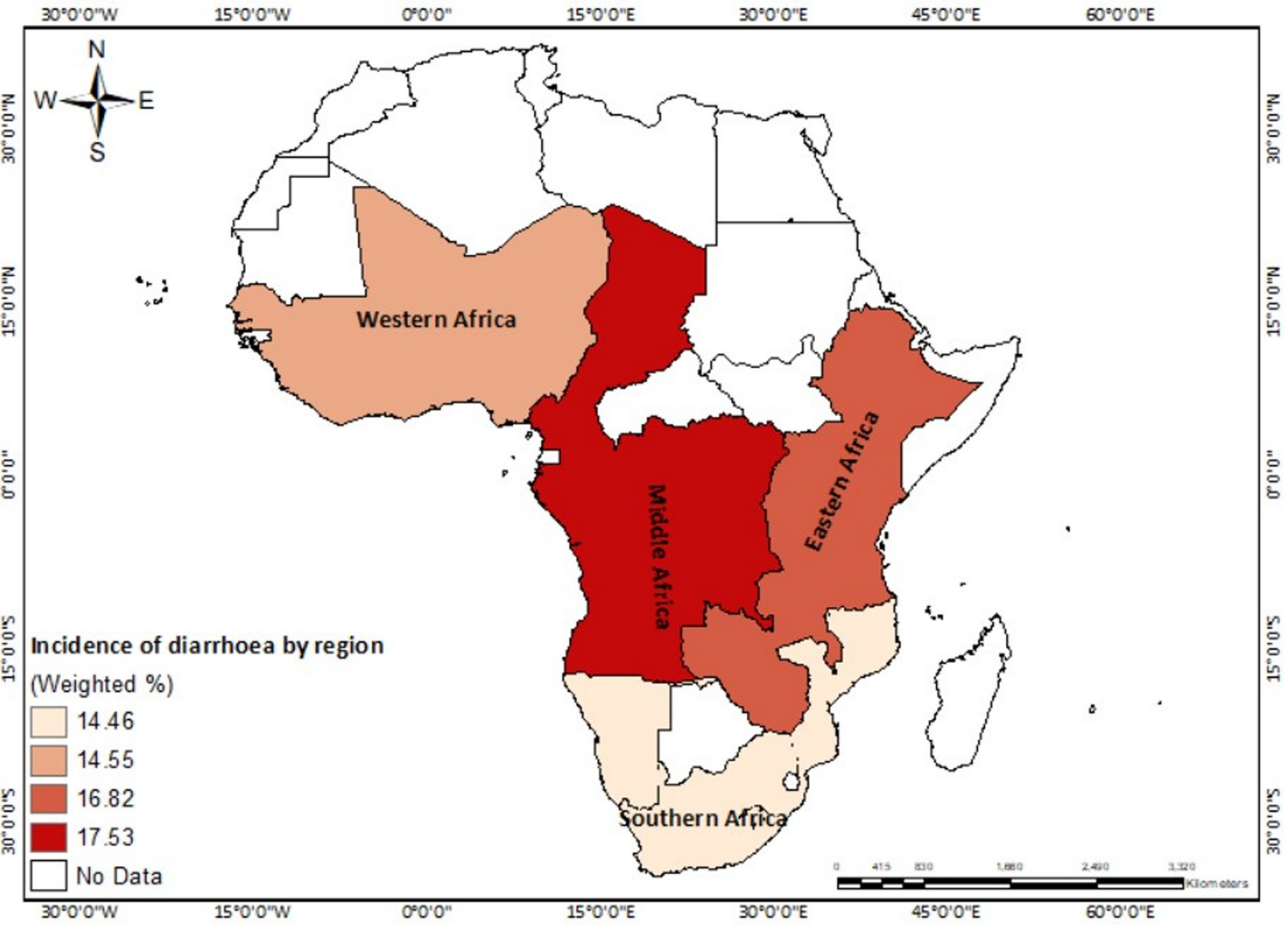
Incidence of diarrhoea among children under five by region of 33 SSA countries. Republished from EfrainMaps, under a CC BY license, with permission from EfrainMaps, original copyright 2022. Note: Authors’ construct based on shapefiles freely downloaded from EfrainMaps website (https://tapiquen-sig.jimdofree.com/descargas-gratuitas/mundo/) and DHS data.

**Fig 4:**
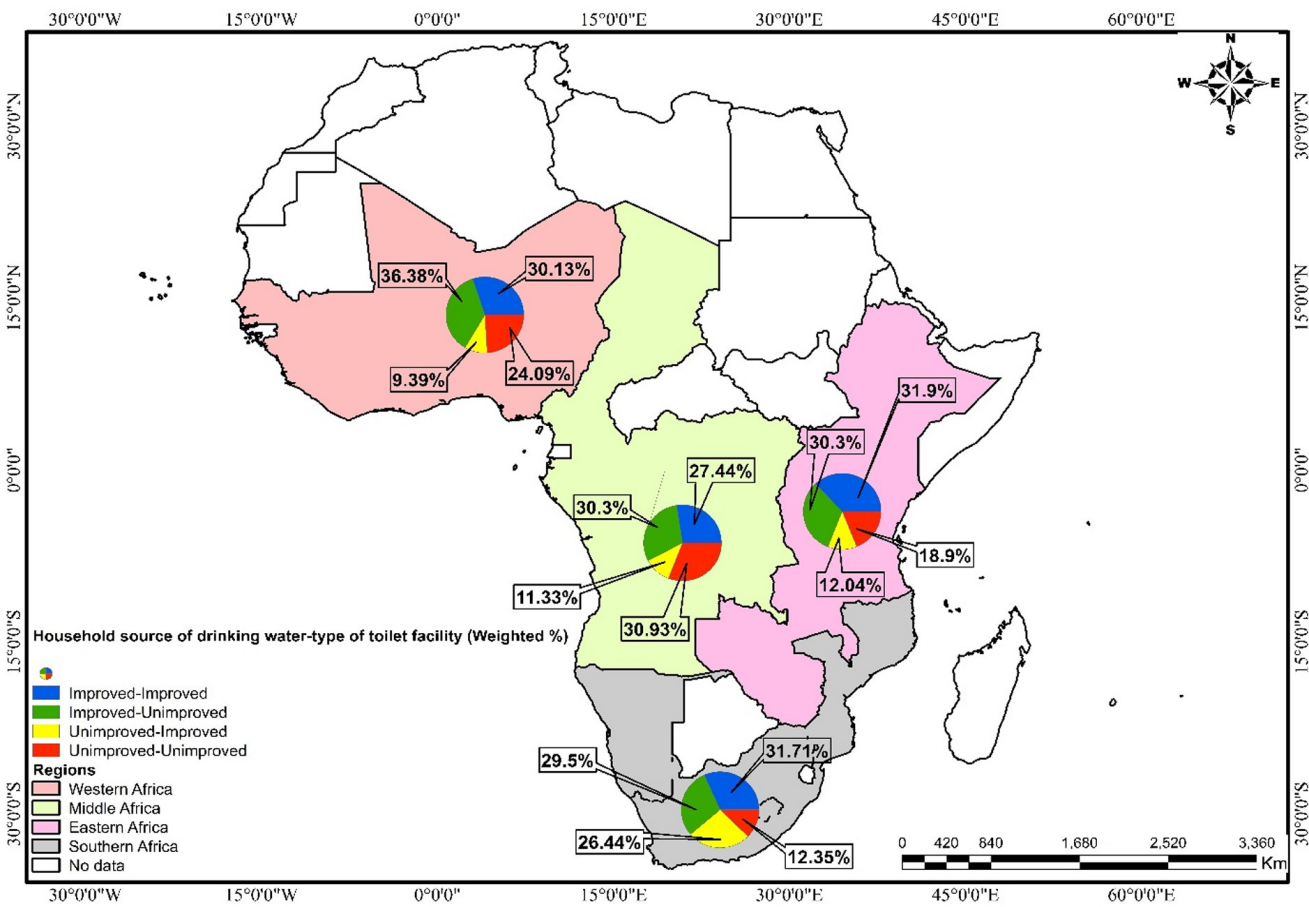
Source of household’s drinking water and type of toilet facility (weighted %) by SSA region. Republished from EfrainMaps, under a CC BY license, with permission from EfrainMaps, original copyright 2022. Note: Authors’ construct based on shapefiles freely downloaded from EfrainMaps website (https://tapiquen-sig.jimdofree.com/descargas-gratuitas/mundo/) and DHS data. Permission has been granted by the copy right owner and it is attached as a S1 Fig.

Of the 307,741 respondents in 33 sub-Saharan African countries, 16% reported diarrhoea among children under five years in their households (Table 1). The prevalence of childhood diarrhoea varied across countries and sub-regions, with Burundi recording the highest prevalence among children under five (22.45%), and Sierra Leone (7.5%) and Benin (10.56%) the lowest (see Fig 2). The spatial autocorrelation results (Moron’s index = -0.175446, z-score = -1.020175, p-value = 0.307645) revealed spatial randomness in the distribution of childhood diarrhoea incidence across the study countries (S1 Fig). This means that the spatial pattern of the high or low incidence of childhood diarrhoea cannot be found around a particular area but randomly distributed across space. For the regional-level analysis, Central Africa had the highest prevalence (17.53%) with the lowest in Southern Africa (14.46%) (Fig 3). In terms of household access to water and sanitation facilities, 32% of all respondents lived in households with an improved source of drinking water and an improved type of toilet facility, while 23% had an unimproved source of drinking water and unimproved type of toilet facility (Table 1). Central Africa had the highest prevalence of unimproved-unimproved sources of drinking water and type of toilet facility (30.93%), whereas Eastern Africa recorded the lowest prevalence of improved-improved source of drinking water and type of toilet facility (31.9%) (Fig 4).

### Descriptive statistics of sample characteristics and relevant variables

Table 2 presents the background characteristics of respondents and relevant variables. About 45% of respondents had a medium-sized household. More than half (51%) of respondents’ children were male. The majority of the respondents had no access to electricity (70%), were poor (45%), lived in rural areas (69%), and had a male-headed household (81%). About 44% of household heads were middle-aged. Most of the mothers of children in the study were within the 25–29 age cohort (27%), had no formal education (41%), delivered at a health facility (64%), had antenatal visits during pregnancy (89%) and did not have postnatal checks within two months (58%). In terms of countries, 10% of respondents resided in Nigeria. The Chi-square test results showed significant associations between all the predictor variables and childhood diarrhoea (Table 3).

**Table 2:** Background characteristics and relevant variables.

Weighted Variable	n	%	Weighted Variable	n	%
**Child had diarrhoea recently**			**Household size**		
No	258,674	84	Small	135,844	41
Yes	49,067	16	Medium	149,070	45
**Source of drinking water-type of toilet facility**			Large	44,972	14
Improved-improved	105,677	32	**Access to electricity**		
Improved-unimproved	110,166	33	No	232,219	70
Unimproved-improved	36,867	11	Yes	97,620	30
Unimproved-unimproved	77,026	23	**Wealth status**		
**Current age of child**			Poor	147,785	45
0	66,161	21	Middle	66,428	20
1	62,432	20	Rich	115,673	35
2	59,647	19	**Urbanicity**		
3	60,949	20	Urban	102,233	31
4	58,775	19	Rural	227,653	69
**Sex of child**			**Country**		
Male	167,312	51	Angola	11,968	4
Female	162,574	49	Benin	12,418	4
**Birth order**			Burkina Faso	14,860	5
1	70,677	21	Burundi	13,472	4
2–4	157,096	48	Cameroon	9,226	3
5 and above	102,113	31	Chad	16,929	5
**Mother’s age**			Comoros	2,605	1
15–19	19,458	6	Congo	7,146	2
20–24	73,744	22	Cote D’Ivoire	6,905	2
25–29	90,189	27	DR Congo	17,470	5
30–34	69,890	21	Ethiopia	10,701	3
35–39	47,915	15	Gabon	4,189	1
40–44	21,991	7	Gambia	7,245	2
45–49	6,700	2	Ghana	5,381	2
**Mother’s highest education**			Guinea	7,467	2
No education	135,456	41	Kenya	18,846	6
Primary	108,179	33	Lesotho	2,725	1
Secondary	75,223	23	Liberia	5,899	2
Higher	11,027	3	Malawi	16,749	5
**Antenatal visits during pregnancy**			Mali	9,629	3
No	23,874	11	Mozambique	5,073	2
Yes	201,441	89	Namibia	3,486	1
**Postnatal check within 2 months**			Niger	12,752	4
No	121,199	58	Nigeria	32,859	10
Yes	88,347	42	Rwanda	7,782	2
**Place of delivery**			Senegal	9,868	3
Home	114,943	35	Sierra Leone	8,454	3
Health facility	208,832	64	South Africa	3,122	1
Other	3,385	1	Tanzania	9,331	3
**Partner/husband’s highest education**			Togo	6,347	2
No education	109,139	38	Uganda	14,012	4
Primary	78,581	28	Zambia	9,017	3
Secondary	78,414	28	Zimbabwe	5,952	2
Higher	18,877	7	**Geographic Region**		
**Sex of household head**			Western Africa	140,084	42
Male	266,611	81	Eastern Africa	113,542	34
Female	63,274	19	Central Africa	66,928	20
**Age of household head**			Southern Africa	9,332	3
Young-adult	143,194	43	N = 329,886		
Middle-aged	144,639	44			
Old	42,043	13			

**Table 3:** Chi-square test results showing the a association between childhood diarrhoea and water and sanitation practices and control variables.

Variable	Had diarrhoea recently (weighted %)	CI	p-value
**Source of drinking water and type of toilet facility**			<0.001
Improved-improved	15.5	15.3–15.7	
Improved-unimproved	16.4	16.1–16.6	
Unimproved-improved	14.8	14.4–15.1	
Unimproved-unimproved	16.5	16.3–16.8	
**Child’s age**			<0.001
0	18.8	18.5–19.1	
1	25.7	25.3–26.0	
2	16.9	16.6–17.2	
3	10.4	10.1–10.6	
4	7.2	7.0–7.4	
**Child’s sex**			<0.001
Male	16.6	16.4–16.8	
Female	15.3	15.1–15.5	
**Birth order**			<0.001
1	16.5	16.2–16.8	
2–4	15.7	15.5–15.9	
5 and above	15.9	15.7–16.2	
**Mother’s age**			<0.001
15–19	22.0	21.4–22.6	
20–24	18.7	18.4–19.0	
25–29	15.7	15.5–16.0	
30–34	14.3	14.0–14.6	
35–39	13.8	13.5–14.1	
40–44	13.1	12.7–13.6	
45–49	12.6	11.7–13.4	
**Mother’s highest education**			<0.001
No education	15.4	15.2–15.6	
Primary	17.6	17.3–17.8	
Secondary	15.5	15.2–15.7	
Higher	10.2	9.6–10.8	
**Antenatal visits during pregnancy**			<0.001
No	17.1	16.6–17.6	
Yes	19.0	18.8–19.1	
**Postnatal check within 2 months**			0.001
No	18.8	18.5–19.0	
Yes	19.2	18.9–19.5	
**Place of delivery**			<0.001
Home	15.9	15.6–16.1	
Health facility	16.1	15.9–16.2	
Other	18.0	16.7–19.4	
**Partner/husband’s highest education**			<0.001
No education	15.4	15.2–15.6	
Primary	17.0	16.7–17.3	
Secondary	16.1	15.9–16.4	
Higher	13.2	12.7–13.7	
**Sex of household head**			0.022
Male	15.8	15.6–15.9	
Female	16.6	16.3–16.9	
**Age of household head**			<0.001
Young	17.0	16.8–17.2	
Middle-aged	14.9	14.7–15.0	
Old	16.2	15.8–16.5	
**Household size**			<0.001
Small	16.4	16.2–16.6	
Medium	15.3	15.1–15.4	
Large	16.9	16.5–17.2	
**Access to electricity**			<0.001
No	16.7	16.5–16.8	
Yes	14.3	14.1–14.5	
**Wealth status**			<0.001
Poor	16.9	16.7–17.1	
Middle	15.5	15.2–15.8	
Rich	15.0	14.8–15.2	
**Urbanicity**			p<0.001
Urban	15.2	15.0–15.4	
Rural	16.3	16.1–16.4	
**Geographic Region**			<0.001
Western Africa	14.6	14.4–14.7	
Eastern Africa	16.8	16.6–17.0	
Central Africa	17.5	17.2–17.8	
Southern Africa	14.5	13.7–15.2	

### Negative log-log regression analysis of childhood diarrhoea and predictor variable relationships

The results (Table 4) from Model 1 showed a significant association between the source of drinking water and type of toilet facility, and childhood diarrhoea. Respondents from households with both unimproved drinking water source and unimproved type of toilet facility were more likely to report childhood diarrhoea (OR = 1.035, 95% CI = 1.020–1.051) than those in households with both improved drinking water sources and type of toilet facility. However, this relationship was mediated by the compositional and contextual factors. When the effects of all the compositional and contextual factors were controlled, respondents from households with an improved drinking water source and unimproved type of toilet facility were more likely to report childhood diarrhoea (aOR = 1.029, 95% CI = 1.015-1-043) than those in “improved-improved” households. After adjusting for all covariates (compositional and contextual factors) in Model 3, living in a household with both unimproved drinking water sources and type of toilet facility was not significantly associated with childhood diarrhoea. However, those from households with an improved drinking water source and unimproved type of toilet facility were still more likely to report child diarrhoea (aOR = 1.020, 95% CI = 1.003-1-036) than those whose households had both improved drinking water source and type of toilet facility. Across the geographical regions, Eastern (aOR = 1.102, 95% CI = 1.084–1.120) and Central Africa (aOR = 1.102, 95% CI = 1.083–1.121) were more likely to experience child diarrhoea.

**Table 4:** Negative log-log regression models showing the relationships between childhood diarrhoea and water and sanitation practices.

Variables	Key predictor					Controlled for compositional factors					Controlled for contextual factors				
	Model 1					Model 2					Model 3				
	cOR	RobustSE	P-value	CI		AOR	Robust SE	P-value	CI		AOR	Robust SE	P-value	CI	
**Source of drinking water-type of toilet facility (Ref: Improved-Improved)**															
Improved-unimproved	1.029	0.007	**<0.001**	1.015	1.043	1.018	0.010	0.072	0.998	1.039	1.020	0.008	**0.018**	1.003	1.036
Unimproved-improved	0.974	0.010	**<0.001**	0.956	0.994	0.983	0.013	0.205	0.957	1.010	0.975	0.011	**0.019**	0.955	0.996
Unimproved-unimproved	1.035	0.008	**<0.001**	1.020	1.051	1.017	0.012	0.148	0.994	1.040	1.017	0.010	0.078	0.998	1.036
**Current age of child (Ref: 0)**															
1						1.251	0.012	**<0.001**	1.227	1.275	1.241	0.010	**<0.001**	1.222	1.260
2						1.006	0.011	0.563	0.985	1.028	1.002	0.009	0.852	0.985	1.019
3						0.828	0.011	**<0.001**	0.807	0.850	0.828	0.009	**<0.001**	0.811	0.845
4						0.729	0.012	**<0.001**	0.707	0.753	0.727	0.009	**<0.001**	0.709	0.745
**Sex of child (Ref: Male)**															
Female						0.953	0.007	**<0.001**	0.939	0.967	0.954	0.006	**<0.001**	0.943	0.966
**Birth order (Ref: 1)**															
2						1.006	0.013	0.652	0.981	1.031	1.001	0.010	0.932	0.981	1.021
5 and above						1.097	0.018	**<0.001**	1.061	1.133	1.090	0.015	**<0.001**	1.061	1.119
**Age of mother (Ref:15–19)**															
20–24						0.993	0.018	0.713	0.958	1.030	0.992	0.015	0.600	0.964	1.021
25–29						0.908	0.018	**<0.001**	0.874	0.943	0.918	0.014	**<0.001**	0.890	0.946
30–34						0.841	0.018	**<0.001**	0.808	0.877	0.854	0.014	**<0.001**	0.827	0.883
35–39						0.804	0.018	**<0.001**	0.769	0.841	0.813	0.015	**<0.001**	0.785	0.843
40–44						0.788	0.020	**<0.001**	0.750	0.829	0.794	0.016	**<0.001**	0.763	0.827
45–49						0.788	0.025	**<0.001**	0.740	0.839	0.801	0.021	**<0.001**	0.761	0.844
**Mother’s highest education (Ref: No formal)**															
primary						1.040	0.011	**<0.001**	1.019	1.062	1.010	0.009	0.245	0.993	1.027
secondary						0.972	0.013	0.028	0.947	0.997	0.942	0.010	**<0.001**	0.922	0.962
Higher						0.848	0.024	**<0.001**	0.802	0.896	0.811	0.018	**<0.001**	0.776	0.847
**Antenatal visits during pregnancy (Ref: No)**															
Yes						1.078	0.014	**<0.001**	1.051	1.106	1.098	0.012	**<0.001**	1.075	1.121
**Postnatal check within 2 months (Ref: No)**															
Yes						1.033	0.008	**<0.001**	1.017	1.049	1.052	0.007	**<0.001**	1.039	1.066
**Place of delivery (Ref: Home)**															
Health facility						0.973	0.009	**0.003**	0.956	0.991	0.967	0.007	**0.000**	0.953	0.981
Other						1.048	0.036	0.179	0.979	1.122	1.056	0.031	0.058	0.998	1.118
**Partner/husband’s highest education(Ref: No formal)**															
Primary						1.056	0.011	**<0.001**	1.034	1.079	1.024	0.009	**0.010**	1.006	1.042
Secondary						1.046	0.012	**<0.001**	1.022	1.071	1.025	0.010	**0.011**	1.006	1.045
Higher						1.019	0.021	0.363	0.979	1.060	1.013	0.016	0.401	0.982	1.045
**Sex of household head (Ref: Male)**															
Female						1.043	0.011	**<0.001**	1.021	1.065	1.021	0.009	**0.017**	1.004	1.038
**Age of household head (Ref: Young)**															
Middle-aged						0.973	0.010	**0.005**	0.954	0.992	0.978	0.008	**0.006**	0.963	0.994
Old						0.973	0.014	0.048	0.947	1.000	0.982	0.011	0.113	0.961	1.004
**Household size (Ref: Small)**															
Medium						1.005	0.010	0.570	0.987	1.024	1.004	0.008	0.631	0.989	1.019
Large						1.061	0.015	**<0.001**	1.033	1.089	1.065	0.012	**<0.001**	1.042	1.088
**Access to electricity (Ref: No)**															
Yes						0.949	0.010	**<0.001**	0.930	0.969	0.977	0.009	**0.008**	0.961	0.994
**Wealth status (Ref: Poor)**															
Middle						0.968	0.010	**0.001**	0.949	0.987	0.967	0.008	**<0.001**	0.952	0.983
Rich						0.998	0.011	0.869	0.977	1.020	0.971	0.009	**0.001**	0.953	0.988
**Urbanicity (Ref: Urban)**															
Rural											0.995	0.008	0.572	0.979	1.012
**Geographic region (Ref: Western Africa)**															
Eastern Africa											1.102	0.009	**<0.001**	1.084	1.120
Central Africa											1.102	0.010	**<0.001**	1.083	1.121
Southern Africa											1.010	0.021	0.610	0.971	1.052

## Discussion

Diarrhoea among children under five years of age is a serious problem in low- and middle-income countries [10]. Studies suggest that improving sanitation practices and access to clean water facilities can help reduce the incidence of diarrhoea among children under five [11–13]. Yet, the joint influence of the drinking water source and sanitation facility on child diarrhoea in SSA is underexplored. Examining the prevalence of diarrhoea disease and its risk factors is critical in developing health interventions to reduce it. Thus, the joint effect of household water source and toilet facility type on diarrhoea prevalence among children under five years old in SSA was explored to facilitate policy action. The prevalence of diarrhoeal disease in SSA among children under five was shown to be 16%, which is high, and has negative impacts on children’s health-related well-being and parents’ cost of health expenditure.

At the country-level, Malawi, Chad, Liberia, and Burundi (22.1–22.5%) recorded the highest prevalence of child diarrhoea among those in the study. Although WHO [14] stressed the use of safe water, handwashing behaviour (using soap), and improved sanitation facilities to prevent child diarrhoea, it is widespread in these countries. Moise (2018) showed that diarrhoea is the third commonest cause of mortality among children under five in Burundi, after malaria and pneumonia. Scholars [15, 16] contend that the lack of access to clean and safe water, limited access to health care, poor sanitation facilities, and undernutrition account for the high incidence of diarrhoea among under-five children. Even though access to health facilities seems to be adequate in Burundi, about 61% of the population cannot afford health care costs [17]. According to OConnell et al. [15], an increased risk of diarrhoea among children under five is associated with unimproved sanitation, younger mothers, and mothers with sub-tertiary education levels. This suggests that public health authorities, sanitation departments, and respective governments may need to provide specific programs targeting increasing access to improved water sources, providing information on hygiene practices, and providing safe water to households both in rural and urban centres. This is critical because, if proper treatment is not given, a higher prevalence of child diarrhoea is likely to result in childhood mortality and morbidity.

Regarding access to improved water and sanitation facilities, 32% of children under-five live in households with access to both improved drinking water sources and toilet facility types. The other 68% may be more likely to report diarrhoea diseases. Following this assumption in this study, a statistically significant association was found between the drinking water source and type of toilet facility, and diarrhoea disease among under-five children. After adjusting for covariates (compositional and contextual factors), the study showed that a household’s drinking water source and type of toilet facility, the child’s current age, sex and birth order, the mother’s age, education, antenatal visits during pregnancy, postnatal check within 2 months, place of delivery, education of partner/husband, household head’s sex and age, household size, access to electricity, wealth status and geographical region within SSA were all significantly associated with the risk of diarrhoea among children under five.

Households with an improved drinking water source and unimproved type of toilet facility, and those with an unimproved source of drinking water and improved type of toilet facility were more likely to report diarrhoea in children under five. This suggests that the risk of diarrhoea increases among children. Due to limited household access to improved drinking water and sanitation facilities in SSA, children under five years are exposed to diarrhoea pathogens that are noted to cause child morbidity and mortality [18, 19]. This is consistent with previous studies showing that households with unimproved toilet facilities and/or drinking water sources were associated with higher child diarrhoea risk [20, 21]. Studies suggest that chlorinating stored water, wells, latrines, and rainwater are appropriate interventions to reduce diarrhoea among children under five, but warn that increased use of chlorine could lead to adverse health outcomes [22, 23]. In some African countries, for example Ghana, the lack of toilet facilities at home has made people, including young children resort to open defecation [24]. No significant association was found in this study between unimproved-unimproved drinking water-type of toilet facility and risk of diarrhoea among children under five, after adjusting for covariates. It could be that households with “unimproved-unimproved” facilities might have opted for favours from neighbours to use their improved toilet facilities, etc. Perhaps those households purchase sachet or bottled water for their children, but this is not known explicitly.

### Strengths and limitations

This study has several strengths. The use of valid surveys and rigorous statistical methods make the findings trustworthy and robust, whilst the use of nationally representative data ensures that the findings are generalisable and replicable in the 33 sub-Saharan African countries included. Moreover, the findings could help bridge gaps identified in current research on childhood diarrhoea, by looking at its association with water and sanitation practices. The study also has some limitations. Since it relied on secondary data, the analyses were limited to variables that were in the datasets. Interpretations and inferences from current findings must thus be limited to those variables observed. Also, the DHS employs cross-sectional designs that restrict causality on the outcomes noted. The key variables were self-reported by the mothers and, therefore, recall bias and other social desirability concerns are likely in the present study.

### Policy and practical implications

Current evidence indicates that the burden of childhood diarrhoea is relatively high in the sub-Saharan African countries, and attributable to inadequate access to clean and safe water, poor sanitation conditions, limited access to health care, wealth, and other socio-economic inequalities. There seem to be substantial geographical variations in the incidence of childhood diarrhoea in SSA, with Malawi, Chad, Liberia, and Burundi having the highest prevalence. The variations in water and sanitation practices among countries could be attributed to disparities in socio-economic and infrastructural development–e.g., housing, social amenities, etc. To address these, governments and relevant stakeholders (e.g., WHO/UNICEF, non-governmental organisations) could adopt strategic approaches aimed at achieving universal and equitable access to improved water and sanitation. For example, introducing initiatives targeted at changing from basic piped or borehole water at the household level to a higher level of service delivery (e.g. regulated, safely managed water supplies) that guarantees safety and health benefits to the population. The various governments should also accelerate actions on providing and improving access to basic sanitation at household level (e.g., flushing to a pit or septic tank, VIP, dry pit latrine with slab, or composting toilet) to help reduce the incidence of childhood diarrhoea.

Strengthening the existing, national WASH program may also contribute to achieving the SDG 2015 Goals– 6: universal and equitable access to improved drinking water and sanitation, and 3: improving health and wellbeing for all. At the community level, behavioural interventions aimed at promoting personal and domestic hygiene should be encouraged, through health education and promotion campaigns as national strategies.

The relationship between education level and diarrhoea infection rates attests to the importance of education in general disease control and health care. Effective diarrhoea control amongst mothers with low education levels should also focus on Behaviour Change Communication, which addresses the knowledge, attitudes and practices of individuals, families and communities, [and] aims to share relevant and action-oriented information and motivate program specialists to work with communication specialists in preparing strategic communication. Similarly, BCC should be incorporated into health education for teenage mothers, to instil behaviour change for effective hygiene practices that will help protect children from disease pathogens.

## Conclusions

This study examined the joint effect of water and sanitation practices on childhood diarrhoea in sub-Saharan Africa. The current reported prevalence (16%) is still high. Water and sanitation practices and geographical region within SSA all contribute significantly to childhood diarrhoea. These findings require multi-sectoral actions that recognise the geo-spatial and temporal characteristics identified, through regional to national policies. Water and sanitation community-based interventions that seek to improve equitable access to safe water and sanitation in the sub-region must be intensified. Moreover, interdisciplinary research aimed at comparing existing interventions (e.g., WASH) using longitudinal designs to assess their effectiveness over time would help policy re-alignment and also pave the way for new strategies.

## Supporting information

S1 Fig (JPG)
