# Supplementary figures and images for: Joint effect of water and sanitation practices on childhood diarrhoea in sub-Saharan Africa
Source: PLoS One. 2023 May 11;18(5):e0283826. doi: 10.1371/journal.pone.0283826 (PMC10174539; doi:10.1371/journal.pone.0283826)

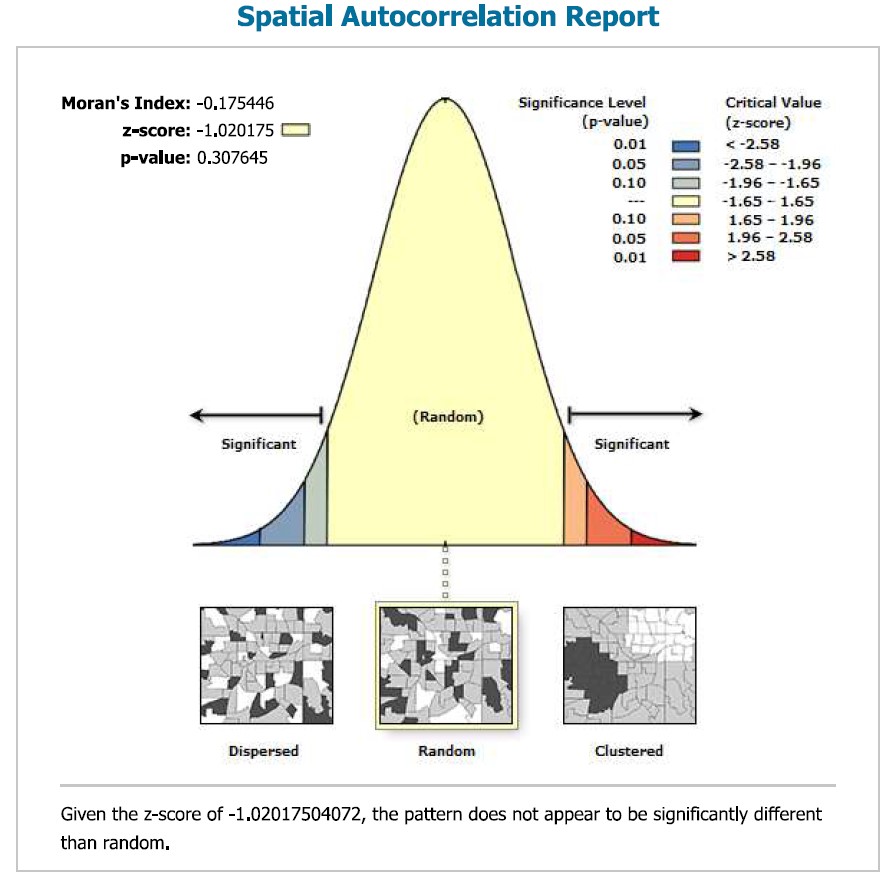

Supplement: S1 Fig — (JPG) [file pone.0283826.s001.jpg]
